# Music Therapy and Tinnitus Treatment: Systematic Review and Meta-Analysis

**DOI:** 10.1055/s-0045-1811695

**Published:** 2026-03-17

**Authors:** Ronaldo Kennedy de Paula Moreira, Maurício Freire Garcia, Patrícia Cotta Mancini, Anna Paula Batista de Ávila Pires, Luciana Macedo de Resende

**Affiliations:** 1 Graduate Program in Speech Therapy Sciences, Universidade Federal de Minas Gerais (UFMG), Belo Horizonte, MG, Brazil; 2 School of Music, Universidade Federal de Minas Gerais (UFMG), Belo Horizonte, MG, Brazil; 3 Speech Therapy Course, Faculty of Medicine, Universidade Federal de Minas Gerais (UFMG), Belo Horizonte, MG, Brazil

**Keywords:** music therapy, acoustic stimulation, tinnitus

## Abstract

**Introduction:**

Tinnitus is the perception of sound without an external source. Various treatments and therapeutic strategies for tinnitus have been used. More recently, this includes music therapy (MT), which aims to mask tinnitus using specific musical elements such as rhythm, melody, harmony, and tempo.

**Objective:**

To identify evidence in the literature regarding the use of MT for the treatment of tinnitus, the techniques employed, and the outcomes achieved.

**Data Synthesis:**

In the present systematic review, we searched for articles in the PubMed, Scopus, Web of Science, MEDLINE, LILACS, and SciELO databases, up until January 2024. The search included articles published in English, Portuguese, and German. Articles on the use of MT for the treatment of tinnitus were included. Duplicates, literature reviews, case reports, letters, and editorials were excluded. The descriptors used were “ *music therapy* ”, “ *acoustic stimulation* ” and “ *tinnitus* ”. Random effects models using the restricted maximum likelihood method were used to estimate the proportion of patients with favorable outcomes. The seardescriptions resulted in the initial selection of 552 articles containing one or more descriptors in the article title. We selected 211 for abstract review, and 48 were selected for full-text review. Finally, 23 articles published between 2005 and 2022 were selected for meta-analysis.

**Conclusion:**

As an effective method for the treatment of tinnitus, MT stimulates the peripheral and central auditory pathways as well as central para-auditory pathways (related to attention, concentration, memory, and emotions).

## Introduction

Tinnitus is the perception of sound without an external sound source and represents a symptom of an underlying condition rather than a single disease. 1 Various treatments and therapeutic strategies for tinnitus have been used, including counseling, tinnitus masking, retraining and reassignment therapies, pharmacotherapy, and, more recently, music therapy (MT). 1 2 Chronic subjective tinnitus has become a growing health issue affecting the quality of life of millions of people worldwide. An increase in individuals with tinnitus has been observed due to the aging population and increased exposure to high levels of noise. 3

The tinnitus retraining therapy (TRT) proposed by Jastreboff is a treatment that includes two main steps: psychological counseling and sound therapy, aimed at reducing the abnormal neural activity. 4 As a newer therapeutic approach, MT is a modification of TRT in which music is used as a sound stimulus. This includes psychoeducation (counseling to reduce negative feelings about tinnitus), singing (especially at frequencies close to or at the specific frequency of the tinnitus), listening to music as a means of distraction, and relaxation techniques. 4 Furthermore, MT aims to mask tinnitus levels, reducing its perception through the use of specific musical elements such as sound, rhythm, melody, harmony, and tempo. 3 Counseling is almost always used in tinnitus treatment, although no study has systematically compared various approaches. When compared to other treatments, no statistically significant difference was found. 2

Currently, there is no standard treatment recommended for tinnitus, and no single protocol. There are several therapeutic options such as pharmacotherapy, acupuncture, neuromodulation, tinnitus masking, psychotherapy, and sound therapy, which can be used alone or in combination. 1 There is no specific drug therapy with proven efficacy for chronic tinnitus; however, it can be useful for treatable comorbidities (e.g., anxiety, depression). 5

Music was first used as a tinnitus masker in 1988, 6 and subsequent studies found it can be used to redirect attention in retraining therapy. However, the existing music used in tinnitus therapies is limited in duration, played repetitively in long-term treatments, and may hinder patient relaxation, even increasing stress levels. Another important aspect is the lack of respect for individuals' preferences, which can compromise treatment. 6 Currently, integrative therapy concepts such as cognitive behavioral therapy (CBT), relaxation therapy, MT, and pharmacotherapy (lidocaine, neurotransmitters) are considered promising therapeutic approaches for tinnitus management. 7

During the construction of this systematic review, our search criteria included the terms “ *Heidelberg Model of Music Therapy (HMMT)* ,” “ *Acoustic Coordinated Reset Neuromodulation (ACRT)* ,” “ *Tailor-made Notched Music (TMNM)* ,” and “ *Fractal Tones (FT)* ”. However, we also observed two other MT techniques, “ *Music and Long Short-Term Memory (MLST)* ” and “ *Auditive Stimulation Therapy (AST)* ”.

**Figure 1:**
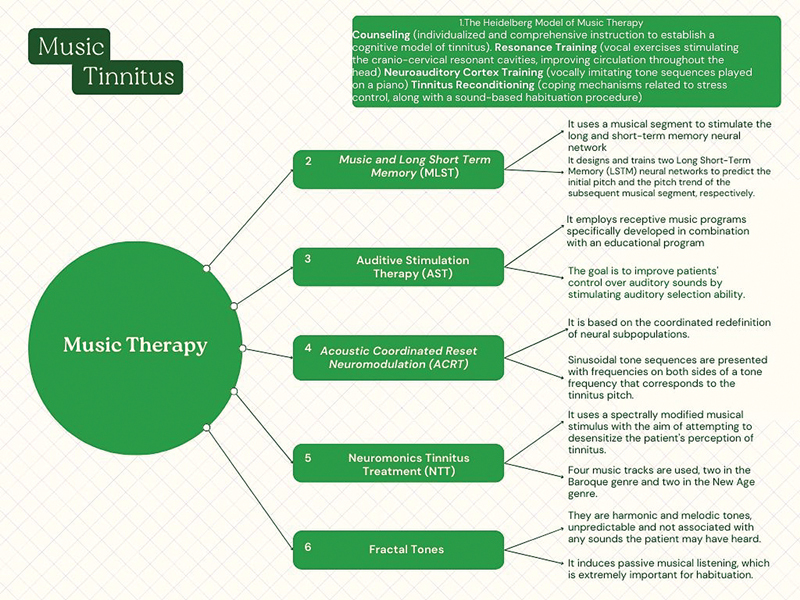
Six types of music therapy techniques.

Regarding MLST, it involves a specific recurrent neural network that can store historical sequence information in its model structure for an extended period. It segments the original music based on the predominant melody pitch sequence and extracts the pitch sequence from each segment of the original music. Specific music is then developed with unlimited and nonrepetitive durations. Patients can receive music that matches their individual preferences and may experience feelings that lead to tinnitus relief after listening. 7 Figure 1 shows a summary of the six MT techniques currently used.

As for AST, it is a complex MT program that employs receptive music programs developed specifically in combination with an education program. Musical self-control training is a program designed based on psychology and MT. The goal is to improve patients' control over auditory stimuli and alleviate feelings of helplessness, stimulating auditory selection ability and reducing unwanted sound perception. 8

## Literature Review

This systematic review sought to answer the following question: “What music therapy techniques have been used for the treatment of tinnitus and what is the degree of effectiveness of these techniques? Do guidelines and updates cite music therapy as a form of tinnitus treatment?” The search strategy was based on Patient, Intervention, Comparison, and Outcome (PICO), which represents the four essential components for constructing the question for bibliographic research.

The present systematic review was conducted following the Preferred Reporting Items for Systematic Reviews and Meta-Analyses Statement (PRISMA) recommendation.

### Search Strategy

Table 1 presents PICO's key components for constructing questions for bibliographic research in the study.

**Table 1:** Key components for constructing questions for bibliographic research in the study: patient, intervention, comparator, and outcome

Questions	Components
Patient	Patients with tinnitus
Intervention	Music therapy for treating tinnitus
Comparator	Tinnitus, music therapy
Outcome	Types of music therapy and relevant effects in tinnitus therapies using music

The descriptors were selected based on consultation with Health Sciences Descriptors (DeCS) and Medical Subject Headings (MeSH), combined with free text terms using the Boolean operator AND. The following combinations were used: “ *Music Therapy* ” AND “ *Acoustic Stimulation* ” AND “ *Tinnitus* ”. There was no restriction on the language of publication.

The electronic databases PubMed, Web of Science, MEDLINE, Scopus, LILACS, and SciELO were searched. After the search, the references from each database were exported to the Mendeley (Elsevier Ltd.) software ( https://www.mendeley.com/ ) to identify all duplicate articles, ensure greater reliability in selection, and proceed to the eligibility stage.

### Eligibility Criteria and Article Selection

Articles met the following criteria to be included in this review: 1) Publications in Portuguese, English, or German; 2) The title should contain the term MT and/or current MT techniques ( *Heidelberg Model of Music Therapy* , *Acoustic Coordinated Reset Neuromodulation* , *Tailor-made Notched Music* , and *Fractal Tones* ), and the word “ *Tinnitus* ” should be present in the title or abstract. Articles that did not mention the MT technique's characteristics or did not describe its results were excluded. Duplicate articles, literature reviews, case reports, letters, and editorials were also excluded.

### Data Analysis

For the analysis of the selected articles, the recommendations of the Strengthening the Reporting of Observational Studies in Epidemiology (STROBE) guidelines were used. During the selection process, after excluding articles that were not within the scope of the present review, the analysis continued by reading the titles and abstracts of the remaining ones.

The articles included were read in full. After reading and analyzing these articles, the selected information included: title, authors, year of publication, country, number of participants, method, description of the technique, results, and conclusions. After the systematic review, 23 articles published between 2005 and 2022 were selected for meta-analysis.

The Heidelberg Model of Music Therapy was first introduced in 2004,. 9 The fundamental concept is based on the idea that tonal tinnitus is experienced as an auditory perception, much like musical stimuli, aiming to integrate the tinnitus into a musically controllable acoustic process. 9 It is composed of four modules: counseling (individualized and comprehensive instruction to establish a cognitive model of tinnitus), resonance training (vocal exercises stimulating cranio-cervical resonant cavities, improving circulation throughout the head), neuro-auditory cortex training (vocally imitating tone sequences played on a piano), and tinnitus reconditioning (coping mechanisms related to stress control, along with a sound based habituation procedure). 9

Maladaptive reorganization of the auditory cortex may contribute to tinnitus generation and maintenance. However, cortical organization can be modified through behavioral training. Tailor-made Notched Music Training exposes patients with chronic tinnitus to enjoyable music that has been modified (“notched”) to exclude the frequency range around the individual tinnitus frequency. Listening to spectrally notched music may reduce cortical activity corresponding to the center frequency, possibly through lateral inhibition. 10 11

Acoustic Coordinated Reset Therapy CR is based on the coordinated redefinition of neuronal subpopulations and was introduced as an effectively desynchronizing stimulation technique, initially used in Parkinson's disease. 12 Noninvasive acoustic stimulation from ACRT reduces the long-term effects of tinnitus annoyance and intensity. Tonal tinnitus mainly arises from abnormal neural synchrony in a tonotopically organized set of neurons. To address this, sinusoidal sequences with frequencies on both sides of a tone corresponding to the tinnitus pitch are presented. 13

Furthermore, FTs are harmonic and melodic tones, unpredictable and not associated with any sounds the patient may have heard. Their unpredictability induces passive musical listening, which is extremely important for habituation (reduces neutral stimuli through repetition). This leads to a state of relaxation and pleasure while listening to the music. Musical elements, such as slower rhythm, lower pitch, degree of repetition, and lack of emotional content, calm people down, taking them out of an alert state. 14

Neuromonics tinnitus treatment (NTT) is an acoustic desensitization protocol that uses spectrally modified musical stimuli to reduce patients' perception of tinnitus. This treatment compensates for hearing loss and volume tolerance across all frequencies by using four music tracks—two in the Baroque genre and two in the New Age genre—delivered through a set of headphones. 1

Some studies combine MT with other tinnitus treatments, such as transcranial direct current stimulation (tDCS), which alters cortical excitability. The prefrontal cortex and auditory cortex are highly sensitive to this stimulation. 15 Other forms of noninvasive brain stimulation, such as transcranial magnetic stimulation (TMS) and transcranial alternating current stimulation (tACS), have also been used. These techniques allow for the evaluation of specific neural structures related to defined cognitive processes like perception, working memory, or attention. 16

The objective of this study was to conduct a systematic literature review and metaanalysis of MT techniques used in current tinnitus treatment approaches.

## Results

Through the search strategies, 552 publications were found (119 in PubMed, 171 in Scopus, 93 in Web of Science, 162 in MEDLINE, and 7 in LILACS). No publications meeting the inclusion criteria were found in the SciELO database.

**Figure 2:**
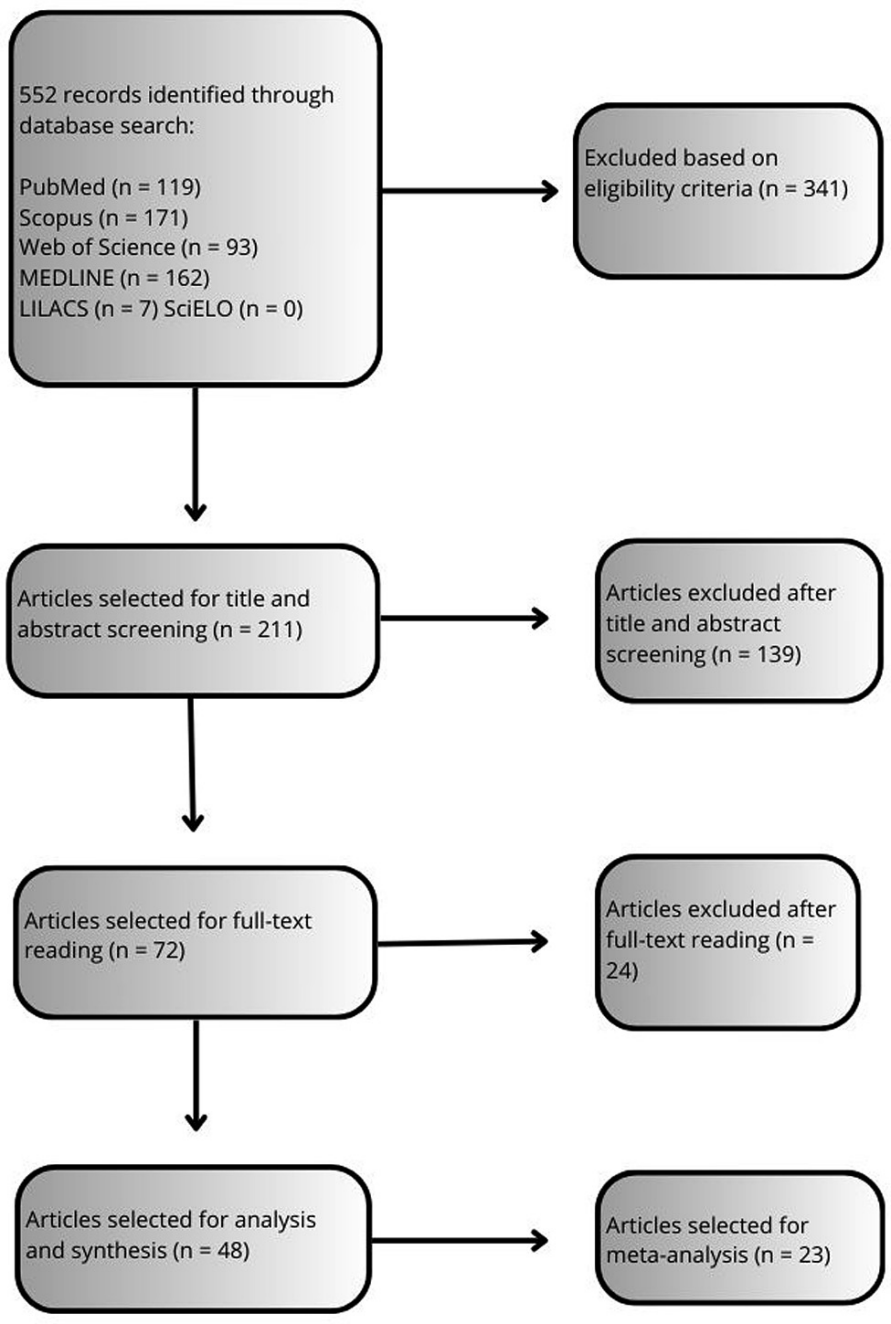
Flow diagram.

Out of those 552 articles, 341 were excluded based on eligibility criteria, leaving 211 with the keywords of this review in their title or abstract. After eliminating 138 duplicates and 1 review article, 72 were selected for full text reading. Afterward, 24 articles were excluded for not containing data on MT as a form of tinnitus treatment. Finally, 48 full-text articles were included in the qualitative analysis, and 23 were selected for the meta-analysis. The entire selection process is described in Figure 2 , which shows the PRISMA 17 flow diagram for article inclusion.

Table 2 presents the synthesis of the 48 studies included in the systematic review. 1 2 3 5 7 8 9 10 11 13 14 15 16 18 19 20 21 22 23 24 25 26 27 28 29 30 31 32 33 34 35 36 37 38 39 40 41 42 43 44 45 46 47 48 49 50 53 54

**Table 2:** Synthesis of the 48 studies included in the systematic review

Authors	Year	Country	Patients (n)	Method	Techniques	Effectiveness	Considerations
Alonso-Valerdi et al. 18	2021	Mexico	108	Non-randomized controlled clinical trial	TRT, TEAE, ADT, music, and TBC	29% (TRT), 19% (TEAE), 14% (ADT), 7% (Music), 14% (TBC)	Based on absolute values, it compared 5 types of music therapy, with no significant effects.
Argstatter et al. 9	2012	Germany	107	Prospective descriptive study	HMMT	76%	A highly effective treatment option with long-lasting effects (5.4 years posttreatment) for chronic tinnitus.
Argstatter et al. 19	2015	Germany	290	Parallel-group controlled clinical trial	HMMT	66% (MT) compared to 33% (counseling)	A comparison between MT and counseling shows better performance with MT.
Argstatter et al. 20	2012	Germany	135	Prospective cross-sectional study	HMMT	80% (MT) 44% (placebo)	It is a quick-onset method with long-lasting effects for patients with ‘tonal’ tinnitus.
Argstatter et al. 21	2009	Germany	40		HMMT	39.3% relative change pre- and post-follow-up	The HMMT is a highly effective and cost-effective method for treating chronic tonal tinnitus, addressing both psychological aspects and neuroscientific methodology.
Argstatter et al. 22	2008	Germany	209	Controlled comparative study	HMMT	80% reduction in tinnitus questionnaire scores. Psychological treatment (21%)	The fMRI was able to provide neural correlation of tinnitus with HMMT.
Argstatter et al. 23	2010	Germany	58		HMMT	87% improvement in symptoms (tonal tinnitus: 71%)	Both tonal and atonal tinnitus should be considered in therapy evaluations.
Attanasio et al. 53	2012	Italy	62	Prospective cross-sectional comparative study	ME	Mozart: 60% improvement, 30% worsening, 10% unchanged.	It compared the efficacy of Mozart and Beethoven music in treating tinnitus, with Mozart showing better performance.
Chen et al. 25	2021	China	60	Nonrandomized clinical trial	MLST	87% (MLST) 50% (counseling)	Preferred music and counseling are effective in treating tinnitus.
Chen et al. 7	2020	China	40	Non-randomized clinical trial	MLST	> 90% of participants experienced positive and relaxed reactions	Reduced the emotional consequences of tinnitus, either as monotherapy or in combination with other MT techniques.
Diao et al. 26	2021	China	70	Non-randomized clinical trial	MNMT	55.1% (TMNMT) 28.3% (control)	Significant reduction in THI score among patients with high scores.
Feng et al. 27	2020	China	56	Prospective comparative study (44) and retrospective comparative study (12)	Preselected music		The combination of music and CBT was more effective than isolated treatments, as supported by EEG.
Goddard et al. 28	2009	USA	32	Non-randomized clinical trial	NTT	78.6% experienced a 40% reduction in TRQ	Significantly reduces the effects of tinnitus on daily life. Future clinical studies are necessary.
Grapp et al. 29	2013	Germany	23	Non-randomized clinical trial	HMMT		
Grapp et al. 30	2013	Germany	15	Non-randomized clinical trial	HMMT	73.3% of patients had a reliable reduction in individual TQ scores.	It proves effectiveness in treating recent tinnitus, but the sample size is small.
Haller M et al 31	2017	England	—	Analysis of articles about therapy	NTT		Controlled trials are more effective for evaluating neuromodulation, with the presence of a placebo group being important.
Hesse et al 32	2007	Germany	20	Prospective controlled randomized study	HMMT	70.0%	Significant and achievable neuronal restructure and reorganization of auditory pathways with this technique.
Hutter E et al 33	2014	Germany	175	Descriptive and retrospective data analysis	HMMT	Considerable overall variability in tinnitus pitch.	A large group of patients experienced a decrease in frequency on a day-to-day basis.
Ibarra-Zarate et al. 34	2022	Mexico	34	Clinical trial	BST	BST reduced stress in 23% of individuals. Tinnitus perception: 23% (both)	BST is recommended for individuals suffering from tinnitus and experiencing stress-related side effects, but not anxiety-related ones.
Kim et al. 54	2017	South Korea	26	Clinical trial	MNMT	THI score improved, particularly in the emotional subscale	This study evaluated the use of TMNMT and *Gingko biloba* in tinnitus treatment through a smartphone application. *Ginggo biloba* amplified the effectiveness of the treatment.
Krick et al. 50	2015	Germany	63	Randomized controlled clinical trial	HMMT		It reports a 16-point reduction in the TQ questionnaire.
Kusatz et al. 8	2005	Germany	155	Prospective observational study	AST	52.3% improvement at the end of therapy	MT is an effective treatment approach and shows progress in tinnitus treatment.
Lan et al. 35	2022	China	86	Clinical trial	MNMT and rTMS	The predictive accuracies were higher in models using FNC measures.	Neuroimaging shows promise in selecting the ideal neuromodulation intervention for tinnitus treatments by identifying connections in brain networks.
Lee et al. 15	2017	South Korea	14	Prospective comparative study	MNMT	The characteristics of tinnitus decreased significantly. 57.1% (annoyance), 42.9% (awareness), 35.7% (impact on life), and 28.6% (tinnitus intensity)	Patients with better contralateral hearing had a higher likelihood of responding to the combination of TMNMT and tDCS.
Li et al. 38	2016	Canada	50	Double-blind randomized clinical trial	MNMT	15-point reduction in THI.	It evaluated the improvement in distress level, suffering, and tinnitus severity. Short-term therapeutic response (3 months).
Low et al. 11	2008	Germany	9	Prospective comparative study	HMMT		ALRs can be used as an objective assessment to quantify the effect of therapies, such as compact MT, for tinnitus.
Moossavi et al. 37	2022	Iran	26	Randomized clinical trial	MNMT		Combined transcranial stimulation and TMNMT, resulting in improvement in tinnitus and cognitive functions.
Newman et al. 3	2012	USA	56	Retrospective clinical study	NTT	NTT and sound generator: 45.0%	Compares NTT and sound generator. Similar results.
Nickel et al. 39	2005	Germany	20	Randomized controlled clinical trial	HMMT	The TQ scores decreased in the pre-post music therapy group by 24.9 points (53%).	Despite the small sample size, the innovative MT produces statistically and clinically significant results.
Nolan et al. 41	2020	Switzerland	268	Retrospective comparative study	Pleasant music		They reinforce the importance of multimodal approaches in treatment (CBT + MT).
Okamoto et al. 10	2010	USA	23	Double-blind longitudinal study	MNMT		The group that received therapy experienced a significant reduction in tinnitus annoyance.
Pantev et al. 40	2014	Germany	100	Double-blind randomized clinical trial	MNMT		First randomized controlled study in a larger number of patients with tonal tinnitus applying TMNMT.
Pape et al. 42	2014	Germany	19		MNMT		Listening to custom-tailored notched music induces greater neuroplastic changes in the maladaptively reorganized cortical network of patients with tinnitus.
Piromchai et al. 2	2021	Thailand	75	Randomized controlled clinical trial	MNMT		Compared TMNMT with conventional MT and counseling. There was no difference.
Shim et al. 43	2015	China	30	Prospective comparative study	MNMT	50% reported relief of symptoms on the global improvement questionnaire	It demonstrated feasibility in bimodal treatment involving vagus nerve stimulation and TMNMT.
Simonetti et al. 14	2018	Brazil	6	Open pilot study	FT	No significant improvements in VAS and good outcomes in functional THI	Benefits of treatment for the functional aspects of THI.
Sruthi et al. 1	2022	India	90	Comparative longitudinal study	Relaxing music		Comparative study, with MT showing better performance than pharmacotherapy.
Stein et al. 45	2015	Germany	9		MNMT		TMNM evokes inhibition-induced plasticity in a distributed network involving temporal, frontal, and parietal junctions.
Stein A et al. 46	2016	Germany	100	Double-blind randomized controlled clinical trial	MNMT		There was an improvement in tinnitus intensity, while measures of tinnitus distress did not show relevant changes.
Sweetow et al. 44	2010	USA	14	Open pilot study	It evaluates the use of a hearing aid, combining amplification, FT, and white noise tone	Improvement in tinnitus annoyance: 86% (fractal tones).	FT may provide relief for some people suffering from tinnitus.
Tass et al. 13	2019	USA	63	Prospective, randomized, single-blind, placebo-controlled study	NTT	75.0% effectiveness	Seeking auditory filters for selecting acoustic tones that incorporate a frequency scale corresponding to the tinnitus pitch.
Teismann et al. 47	2011	Germany	24	Comparative study of matched groups	MNMT		Short-term intensive TMNMT reduced subjective tinnitus intensity on patients with frequencies ≤ 8 kHz.
Teismann et al. 16	2014	Germany	32	Prospective study	MNMT		The short-term combined treatment of rTMS + TMNMT can reduce discomfort related to tinnitus.
Therdphaothai et al. 5	2021	Thailand	108	Randomized controlled clinical trial	MNMT		Positive (median) results with low statistical significance.
Argstatter at al. 24	2007	Germany	20	Prospective controlled study	HMMT	In the pre-post comparison, values in the therapy group decreased by 53%, and 5% in the control group	There was a significant reduction in the degree of tinnitus annoyance in the treatment group compared to the control group.
Williams et al. 48	2015	Germany	66	Open observational study	ACRT	Average reduction of 25.8% in tinnitus intensity and 32% in tinnitus annoyance	Could not reach an absolute conclusion regarding the effectiveness of the therapy, due to the lack of a control group.
Wunderlich et al. 49	2015	Germany	28	Double-blind randomized clinical trial	MNMT	The average reduction in tinnitus distress was 11%.	Listening to notched music leads to a reduction in neural activation in the notched frequency area.
Yoo et al. 36	2022	South Korea	90	Double-blind randomized prospective clinical trial		THI ≥ 20% reductions were 78.0% and 78.8% in the experimental and control groups, respectively, at 3 months, and 69.2% and 86.7%, respectively, at 6 months	It did not prove cortical reorganization due to inadequate technique.

**Abbreviations:** ACRT, acoustic coordinated reset meuromodulation; ADT, auditory discrimination therapy; ALRs, auditory late responses; AST, auditive stimulation therapy; BBT, binaural beats therapy; BST, binaural sound therapy; CBT, cognitive behavioral therapy; EEG, electroencephalography; fMRI, functional magnetic resonance imaging; FNCs, functional network connections; FT, fractal tones; HMMT, Heidelberg model of music therapy; ME, Mozart effect; MLST, music and long short term memory; MT, music therapy; NTT, neuromonics tinnitus treatment; tACS, transcranial alternative current stimulation; tDCS, transcranial direct current stimulation; TEAE, therapy for enriched acoustic environment; THI, Tinnitus Handicap Inventory; TMNM, Taylor-made notched music; TQ, Tinnitus Questionaire; TRQ, Tinnitus Reaction Questionnaire; TRT, tinnitus retraining therapy; tVNS, transcutaneous vagus nerve stimulation.

The variables language, country of origin, and study design were described to aid in the characterization of studies included in the review but were not part of the main outcomes.

Table 3 ( Supplementary Material Table ) shows the number of articles per country. Germany had the highest scientific production on MT, with HMMT being the most studied.

**Table 3:** Article production by country of origin

Country	Number of studies n (%)
Germany	22 (45.8%)
Brazil	2 (4.2%)
Canada	1 (2.1%)
China	6 (12.5%)
South Korea	3 (6.1%)
USA	5 (10.4%)
England	1 (2.1%)
India	1 (2.1%)
Iran	1 (2.1%)
Italy	1 (2.1%)
Mexico	2 (4.1%)
Switzerland	1 (2%)
Thailand	2 (4.2%)
**TOTAL**	**48 (100%)**

The 48 selected articles were published between 2005 and 2022, with 44 having been published in English, 3 in German, and 1 in Portuguese.

The MT techniques that had the most published articles was TMNM, with 18, 2 5 10 15 16 18 26 34 35 37 38 39 42 43 44 45 46 50 followed by the HMMT, with 13. 9 11 19 20 21 22 23 29 30 32 33 40 50 Response rates to treatments were significant in most articles that used these two types of MT. 9 10 15 19 20 22 23 26 32 37 40 However, some reported median results with low statistical significance (< 5), as well as inconclusive results. 16

The largest sample size in the studies, 19 ranging from 6 to 290 individuals, was conducted in comparison between MT and counseling, with better performance in MT results. Other studies compared the efficacy of different tinnitus treatment techniques, 1 2 3 19 51 and one compared the effectiveness of the HMMT, tinnitus maskers, and pharmacotherapy ( *Gingko biloba* and antioxidants), with MT showing better performance. 1 Most articles reported good performance of MT in tinnitus treatment. One study comparing TMNMT, conventional MT, and counseling did not find statistically significant differences in the outcomes. 2 Articles also described the advantages of bimodal therapy, showing improved therapeutic results by combining MT with cognitive-behavioral therapy, 27 TMS, 39 and vagus nerve stimulation. 44

### Meta-Analysis

The meta-analysis is a statistical technique specially developed to integrate the results of two or more independent studies on the same research question, combining the results of such studies into a summary measure or even reaching a new conclusion from a systematic literature review. 52 The meta-analysis of proportions aims to obtain a more precise estimate of the overall proportion of a certain event.

The objective of the present study was to evaluate the proportion of patients with favorable outcomes from MT treatment for tinnitus. There were 23 articles, published between 2005 and 2022, selected and included in our analysis. 3 7 8 9 13 15 18 19 20 21 22 23 26 28 32 34 36 37 40 44 47 51 53

To estimate the proportion of patients with favorable outcomes, random effects models using the restricted maximum likelihood method (REML) were employed. The Q test was used to identify heterogeneity among studies, and the I2 statistics to quantify this heterogeneity. Publication bias was assessed using funnel plots and a linear regression-based asymmetry test.

To check for the presence of outliers, data points that significantly differ from all others, externally studentized residuals were used. Outlier studies are not necessarily influential, meaning they don't significantly change parameter estimates. The leave one-out technique was used to detect influential studies. In our analysis, none of the studies were identified as outliers or influential. A significant level of 5% was adopted.

**Figure 3:**
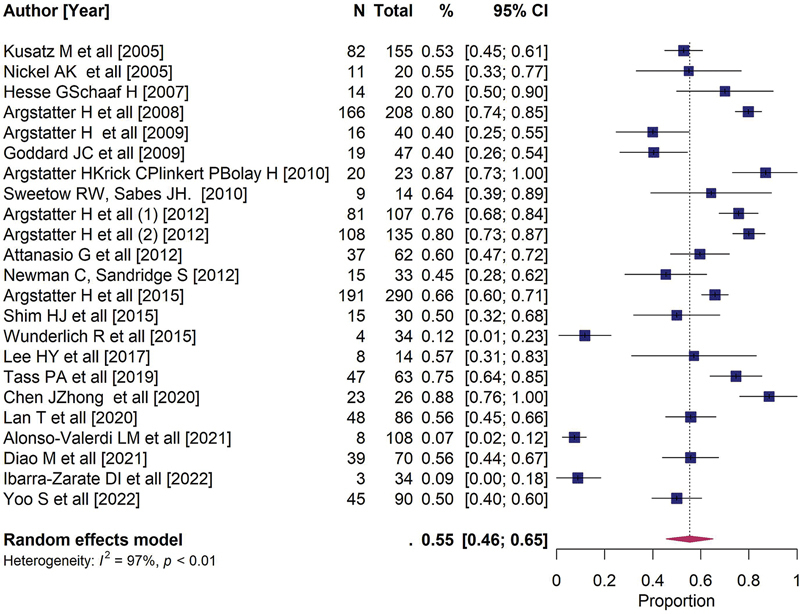
Summary of the meta-analysis to estimate the prevalence of favorable outcomes.

Figure 3 presents a summary of the meta-analysis to estimate the prevalence of favorable outcomes.

From the 23 studies, data from a total of 1,709 participants were obtained, with an estimated percentage of 55% and a 95% confidence interval (CI) between 46 and 65%. High heterogeneity among the articles was observed, as evidenced by both the Q test ( *p* < 0.01) and the I2 statistic (97%).

The forest plot is a graph that allows visualization of the estimated measures and their CIs. Each study is plotted on the graph with two elements: a box representing the estimate of each study and a horizontal line representing that estimate's CI. In a qualitative analysis, the articles' relevance was similar, as the size of the boxes was visually similar. Smaller horizontal lines indicate better precision of the results (narrower CIs). Authors such as Alonso-Valerdi et al., 18 Argstatter et al., 22 and Newman et al. 3 had higher levels of precision in their studies, while Lee et al. 15 and Sweetow et al. 44 had lower precision.

The diamond shape represents the estimated effect and its CI. The estimated random effects of treatment described in articles 8 15 26 40 44 were around 55%, with varying CIs. There were 11 articles (47.8%) 7 9 13 15 19 20 22 23 32 47 53 with effect estimates above the estimated random effects. The magnitude of heterogeneity among the studies was very high (I2 = 97%, *p* < 0.01), making it difficult to combine the studies' results. Therefore, we cannot explain their differences due to sampling errors but, likely, due to bias or methodological differences.

One type of bias happens when the probability of study publication depends on its results. Meta-analysis techniques allow unbiased estimates of the population's average effect size. However, if the sample itself is biased, the estimate won't be representative of the population. The funnel plot is a scatter plot of the observed effect against the study's variation. In the absence of publication bias, the points on the graph are symmetric, forming a funnel shape. Studies with lower variability should cluster around the estimated effect. In the lower part of the graph, with higher variability, studies are expected to be more spread out from the estimated effect. Smaller sample sizes may indicate higher variability, but there can be (although rare) studies with large sample sizes and high variability, which also places them at the base. In the present study, symmetry was observed in the funnel plot ( *p* = 0.695).

Articles positioned outside the funnel area 7 13 18 20 22 23 34 41 44 51 are studies with estimates and/or variations different from expected, but this difference was not enough to consider asymmetry.

**Figure 4:**
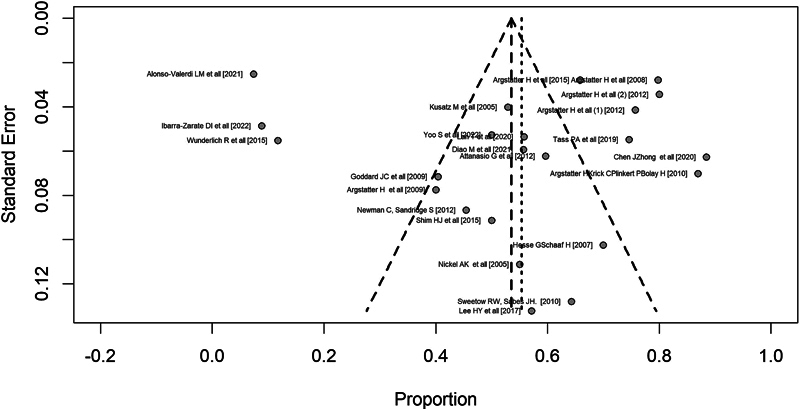
Funnel plot of the studies included in the meta-analysis.

Figure 4 presents the funnel plot of the studies included in the meta-analysis.

## Discussion

The systematic review presented here indicates that MT is widely used in the treatment of neurological diseases, physical, and mental disorders, being considered an effective therapeutic approach for treating tinnitus. 1 19 40 51 By integrating tinnitus with music, it can make the symptom musically controllable. Consequently, tinnitus is no longer perceived as a disturbing sound but rather as music. 8 32 40

Interestingly, one study contradicted the use of sound-based therapies for tinnitus treatment, 18 going against the majority that showed positive results with varying degrees of effectiveness. It compared four sound-based therapies with music (notch therapy) and found slightly positive but not significant results. The authors reported numerous factors influencing study outcomes (both negative and positive) and questioned the oversight of participants' individual characteristics and needs. 18 Another two studies did not show significantly positive results; however, they did not oppose MT either. 18

In this systematic review, we observed a variety of assessment models across different applications of MT. There is no uniformity in methodology types. Studies employ various designs, such as prospective comparative, 7 23 24 27 28 31 33 39 clinical trials, 2 5 18 25 26 31 46 51 and descriptive, 39 54 among others.

We also noted a wide variation in sample sizes (from 6–290 participants), with studies reporting favorable results for MT in tinnitus treatment, albeit with small sample sizes. 13 30 32 40 46 52 Several questionnaires were used to assess discomfort levels, including the Tinnitus Handicap Inventory (THI), 1 2 3 5 14 15 36 38 44 47 51 52 Tinnitus Fragebogen (TF), 54 Tinnitus Questionnaire (TQ), 8 22 55 Tinnitus Reaction Questionnaire (TRQ), 9 11 37 40 47 50 Visual Analogue Scale (VAS), 1 14 44 and Tinnitus Handicap Questionnaire (THQ), 46 49 either alone or in combination. There is a lack of standardization in the use of these questionnaires. This diversity in study construction practices makes it challenging to reach a consensus on an MT technique that can serve as a reference for comparing the effectiveness of others.

Only three studies compared MT's effectiveness in tinnitus treatment with other treatment options, 2 3 51 showing better therapeutic performance for the first. So far, we have not identified studies that prove effectiveness through a large-scale controlled clinical trial establishing an active comparison rather than placebo. However, despite this diversity in scientific production possibilities, MT has shown benefits in tinnitus treatment.

With advancements in neuroscience, neurophysiological investigation methods become crucial for research as they enable the exploration of the neurobiological effects of MT on tinnitus-related neuronal correlations. Techniques such as functional magnetic resonance imaging (fMRI), magnetoencephalography (MEG), and electroencephalography (EEG) are particularly important in this context. Studies have utilized fMRI to assess the neuronal correlations of MT, 21 23 50 identifying increased neuronal activities in specific brain areas following such stimuli (frontoparietal network, anterior insula, prefrontal cortex, auditory cortex). Researchers like Argstatter et al. 22 have reported clear indications of tinnitus-related neuronal correlations in patients using fMRI, with pre- and post-therapy measurements.

These studies support the tinnitus model, which suggests this is not solely a symptom generated by auditory dysfunction (bottom-up theory) but also involves a top-down mechanism where nonauditory brain structures play a central role. 22 23 The use of MEG allows the measurement of magnetic fields associated with brain electrical activity, pinpointing functional regions of the cerebral cortex (spontaneous and sensory-evoked activity). 56 Stein et al. 46 have observed reduced neural activity evoked by tinnitus pitch in temporal, parietal, and frontal regions within the N1m time interval using MEG. Other studies 34 43 48 have also used MEG to evaluate the outcomes of their MT techniques.

Feng et al. 27 used EEG to assess the results of comparative studies between cognitive behavioral therapy (CBT) and MT, showing increased powers in alpha and theta bands after MT-CBT treatment and increased gamma power after CBT. An important aspect of tinnitus treatment is the possibility of combining techniques to enhance the outcome. However, there aren't many studies in this area. A definitive treatment with long-lasting effects targeting the multiple neural pathways of tinnitus generation hasn't been found yet.

Combining one or more therapies may improve the quality of tinnitus treatment. Two studies combined TMNMT and CBT for tinnitus treatment, both noninvasive neuromodulation methods. 39 48 These authors demonstrated that the combination of CBT-TMNMT on the dorsolateral prefrontal cortex can reduce discomfort levels. Another study combined TMNMT and transcutaneous vagus nerve stimulation (tVNS) for tinnitus treatment. During tVNS, patients listened to notched music (TMNM), and about 50% of them reported an improvement in discomfort levels.

Additionally, Feng et al. 27 tested the efficacy of an integrative treatment for tinnitus combining MT and CBT, showing an improvement in tinnitus discomfort levels with the TMNMT-CBT combination after three months of intervention. Nolan et al. 41 demonstrated a significant reduction not only in tinnitus and hyperacusis symptom intensity but also in the resulting discomfort. These findings highlight the potential benefits of combining therapies in tinnitus management, showcasing the importance of exploring multimodal approaches for better treatment outcomes.

## Conclusion

As sound-based therapies for tinnitus treatment, especially MT, can become highly effective, always considering therapy customization for each individual, applied at the right time and in the appropriate context for the patient. The meta-analysis in this review showed a very high heterogeneity among the articles, likely due to methodological differences. However, the effectiveness of the techniques demonstrated positive results of varying degrees in most studies. There is a need for ongoing research to correct methodologies and assess whether a single treatment or multiple simultaneous or sequential treatment methods are necessary to make MT more effective.

There is the possibility of combining treatments to enhance the outcome of tinnitus treatment. In this case, MT can be used either as a standalone therapy or in combination with other treatments like TMNT. It presents a low risk of permanent hearing loss, especially when using silent coil devices and proper hearing protection, proving effective across various socioeconomic levels, although access may be limited for some. Side effects are generally mild, including headaches and local discomfort. The duration of treatment typically ranges from 4 to 7 weeks and can be personalized.

Music is part of our cultural heritage, being experienced daily, even inside the uterus. The way we integrate sounds into our daily lives is significant, and to use it as a therapeutic procedure deeply integrates it into our everyday lives. This aspect of MT can contribute significantly to its effectiveness and acceptance as a treatment modality for tinnitus and other conditions.

## Final Comments

The present systematic review led us to conclude that MT techniques for the treatment of tinnitus have been studied worldwide. Techniques such as HMMT, TMNM and ACRT have shown promising results. More studies are needed, especially clinical trials, with more uniform methodologies, to consolidate the techniques for the treatment of tinnitus.
