# Supplementary material for: Music Therapy and Tinnitus Treatment: Systematic Review and Meta-Analysis
Source: Int Arch Otorhinolaryngol. 2026 Mar 17;30(1):1–13. doi: 10.1055/s-0045-1811695 (PMC12995452; doi:10.1055/s-0045-1811695)
Supplement: Supplementary file 1 — Supplementary Material [file 10-1055-s-0045-1811695-s241785.pdf]

Supplementary Table - Checklist items to be included in the systematic review or meta-analysis report

Section/Topic	N	Checklist Item	Reported on Page
TITLE			
Title	1	Music therapy in the treatment of tinnitus: systematic review and meta-analysis	1
SUMMARY			
Structured summary	2	Tinnitus is a common complaint that affects the quality of life of its sufferers. Music therapy is an excellent treatment option for tinnitus.<br>We searched the PubMed, Scopus, Web of Science, MEDLINE, LILACS, and SciELO databases for articles published in English, Portuguese, and German that described music therapy as a treatment for tinnitus.<br>The search strategy resulted in an initial selection of 552 articles that contained one or more descriptors in the title. From these, 211 articles were selected for abstract reading, and 48 articles for full-text reading. All types of statistical analyses were considered, and 23 articles published between 2005 and 2022 were selected for the meta-analysis.<br>Music therapy proved to be a highly useful therapeutic option in the treatment of tinnitus, especially tonal tinnitus, by stimulating the peripheral and central auditory pathways, as well as the central para-auditory pathways (attention, concentration, memory, and especially emotions).	2 e 3
INTRODUCTION			
Rational	3	Chronic tinnitus is a growing health problem due to the aging population and exposure to loud sounds. There are various therapeutic options for treating tinnitus, used either alone or in combination, but there is no standard treatment. Music therapy is a therapeutic option used worldwide, motivating us to research the literature on musical techniques used for the treatment of tinnitus.	4-8
Objectives	4	"What music therapy techniques have been used for the treatment of tinnitus and how effective are these techniques?"<br>The fundamental components for constructing the questions for the bibliographic search in the research were: Patient (tinnitus sufferers), Intervention (music therapy), Comparator (tinnitus, music therapy), and Outcome (types of music therapy and relevant effects in tinnitus therapies).	9-10
METHOD			
Protocol	5	This systematic review was conducted following the recommendations of the Preferred Reporting Items for Systematic Reviews and Meta-Analyses Statement (PRISMA).	9
Eligibility criteria		The descriptors were selected based on consultation with DeCS (Health Sciences Descriptors) and MeSH (Medical Subject Headings), combined with free terms, using the boolean operator AND. The combinations used were: "Music Therapy" AND "Acoustic Stimulation" AND "Tinnitus." Initially, there was no restriction on the language of publication, with a search for articles published between 2005 and 2024.	
Information sources	6	The databases used were PubMed, Scopus, Web of Science, MEDLINE, LILACS, and SciELO, up until January 2024. The latest article found was a systematic review and meta-analysis of articles on the Heidelberg model of music therapy and Tailor-made Notched Music.	10
Search	7	The first search was conducted on PubMed. Initially, the search focused on titles using the following combinations: "Music Therapy" (OR "Heidelberg Model of Music Therapy" OR "Acoustic Coordinated Reset Neuromodulation" OR "Tailor-made Notched Music" OR "Fractal Tones") AND "Tinnitus."<br>Subsequently, the abstracts of the articles were read, selecting those that discussed music therapy technique(s) and the results obtained with the treatment. Full texts of the selected articles were then read.	11-12
Study selection	8	After reading the abstracts of the initially selected articles, those that discussed a music therapy technique and the results obtained with the treatment were selected for full-text reading.	12
Data collection process	9	During the reading of the articles, we selected relevant parts of the text in a summarized form and then analyzed the summaries to compose the text. We then created a summary table with the 48 studies included in the systematic review.	12

 © 2026. The Author(s).

Supplementary Table (*Continued*)

Section/Topic	N	Checklist Item	Reported on Page
List of data	10	The data list was composed of the author's name, country of publication, year, the music therapy technique used, and an evaluation of the results of the technique used, considering comparisons between tinnitus treatment techniques and other relevant considerations.	12
**RESULTS**			
Study selection	11	A total of 48 studies were selected for analysis, with systematic reviews excluded, even though the full articles were read.	12
Study characteristics	12	All studies were analyzed to primarily determine the effectiveness of the treatment, regardless of sample size or method used, as long as it allowed for discussion.	17
Risk of bias in each study	13	It has not been evaluated.	
Summary of results	14	They were described in the meta-analysis.	19
Risk of bias between studies	15	They were described in the meta-analysis.	19
**DISCUSSION**			
Summary of evidence	16	Despite its effectiveness as a therapy for tinnitus, music therapy is rarely studied and underutilized in our context. It is widely used for neurological disorders, with significant results. The ultimate goal is to draw attention to the use of music therapy in the treatment of tinnitus, considering that tinnitus is a symptom of neurological origin.	25
Limitations	17	The meta-analysis revealed a high degree of heterogeneity among the articles, likely due to methodological differences, making it difficult to draw more concise conclusions.	25
Conclusions	18	Sound-based therapies for the treatment of tinnitus (e.g., music therapy) can become highly effective when applied at the right time and in the appropriate context for the patient. Music is part of our cultural heritage, being an experience lived daily, starting from intrauterine life. We experience listening to various sounds significantly in our daily lives, and we would be using a therapeutic procedure that is extremely integrated into our everyday routine.	25
**FUNDING**			
Funding	19	There was no funding.	25

 © 2026. The Author(s).
